# Mycophenolate Mofetil for Treatment of Ipilimumab-Induced Colitis in Patients with Metastatic Melanoma

**DOI:** 10.3390/cancers18050734

**Published:** 2026-02-25

**Authors:** Christina Naoum, Julia K. Winkler, Pawel Majenka, Carmen Loquai, Annika Brekner, Milena Fuchß, Katharina C. Kähler, Marilena Petersen, Ralf Gutzmer, Imke von Wasielewski, Jessica C. Hassel

**Affiliations:** 1 Department of Dermatology and National Center for Tumor Diseases (NCT), Medical Faculty Heidelberg, Heidelberg University, NCT Heidelberg, A Partnership Between DKFZ and University Hospital Heidelberg, 69117 Heidelberg, Germany; julia.winkler@med.uni-heidelberg.de (J.K.W.);; 2 Department of Dermatology, University Medical Center, Johannes-Gutenberg-University, 55122 Mainz, Germany; carmen.loquai@fachklinik-hornheide.de (C.L.); annika.brekner@unimedizin-mainz.de (A.B.); milena.fuchss@unimedizin-mainz.de (M.F.); 3 Department of Dermatology, Fachklinik Hornheide, 48157 Munster, Germany; 4 Department of Dermatology, Campus Kiel, University Hospital Schleswig-Holstein, 24105 Kiel, Germany; katharina.kaehler@uksh.de (K.C.K.);; 5 Department of Dermatology, Johannes Wesling Medical Center Minden, Campus Minden, Ruhr University Bochum, 44801 Bochum, Germany; ralf.gutzmer@muehlenkreiskliniken.de; 6 Department of Dermatology and Allergy, Skin Cancer Center Hannover, Hannover Medical School, 30625 Hannover, Germany; vonwasielewski.imke@mh-hannover.de

**Keywords:** checkpoint inhibitor induced colitis, steroid-refractory colitis, mycophenolate mofetil, infliximab, metastatic melanoma, immune-related adverse event, ipilimumab

## Abstract

**Background/Objectives:** Treatment with immune checkpoint inhibitors (ICIs) in patients with advanced melanoma has greatly improved clinical outcomes but can induce immune-related adverse events (irAEs). ICI-induced immune-related (ir) colitis is one of the most common severe irAEs and is primarily managed by treatment with high-dose corticosteroids. Some patients are steroid-refractory and require additional immunosuppressants. Infliximab is commonly used as the additional treatment of choice, while data for other immunosuppressants such as mycophenolate mofetil (MMF) are sparse. **Methods:** We used MMF to treat ir colitis in a cohort of patients in Heidelberg and compared clinical data with patients who received standard irAE management with infliximab in the Cancer Centers of Heidelberg, Hannover, Mainz, and Kiel. Outcome measures included response rate, time to response, duration and amount of steroid intake, and recurrence of colitis, as well as progression-free survival (PFS) and overall survival (OS) measured from the start of steroid intake. **Results:** Out of 52 patients refractory to steroids, 31 were treated with additional MMF and 21 with additional infliximab. A total of 24 out of 31 patients (77.4%) experienced bowel habit normalization during treatment with MMF after a median of seven days. Seven patients required additional infliximab to achieve a resolution of symptoms. Twenty out of 21 patients (95.2%) showed a normalization of stool frequency with infliximab after a median of eleven days. One patient required additional MMF to achieve a normal bowel habit. Hence, resolution of symptoms was achieved during both treatment regimens (*p* = 0.081) in a comparable period of time (*p* = 0.858). Neither recurrence of colitis after additional immunosuppression (*p* = 0.760) nor rate of CMV positivity after recurrence of colitis (*p* = 0.898) differed between groups. We observed a tendency towards longer treatment duration (108 vs. 85 days, *p* = 0.052) and significantly higher cumulative corticosteroid intake (7585 mg vs. 3485 mg, *p* = 0.002) in the MMF group compared to the infliximab group. However, we observed significantly higher cumulative steroid intake and a longer duration of corticosteroid therapy in patients treated at the Heidelberg center compared with those treated at the other participating centers within the infliximab group. In contrast, no significant differences in corticosteroid duration or cumulative dose were observed in the center-internal comparison between MMF- and infliximab-treated patients at Heidelberg. These subgroup analyses may indicate that the observed differences in corticosteroid exposure are more likely related to center-specific management strategies rather than substance-specific effects. Neither median PFS nor OS differed between the groups (mPFS: MMF: 3.2 months; infliximab: 2.1 months (*p* = 0.978); mOS MMF: 12 months; infliximab: 9.5 months (*p* = 0.561)). **Conclusions:** The data point to MMF as a well-tolerated, oral treatment alternative for patients with ICI-induced ir colitis, especially in patients where infliximab is contra-indicated.

## Simple Summary

Treatment with immune checkpoint inhibitors (ICIs) has improved survival in patients with advanced melanoma but can also induce immune-related (ir) adverse events such as severe colitis. Steroid-refractory patients are most commonly managed with additional infliximab, while data for other immunosuppressants are sparse. In this retrospective study, we compared mycophenolate mofetil (MMF) with infliximab in patients with steroid-refractory ir colitis. Our study demonstrates that patients can be effectively managed not only with infliximab but also with MMF, without prolongation of time to resolution or impairment of oncological outcomes. The data point to MMF as a well-tolerated, oral treatment alternative for patients with ICI-induced ir colitis, especially in patients where infliximab is contra-indicated.

## 1. Introduction

Melanoma remains a potentially fatal malignancy, and its incidence continues to increase [1,2]. While most patients present with localized disease that can be cured by surgical excision, a considerable proportion will eventually develop metastases. For these patients, immune checkpoint inhibitors (ICIs) such as anti-PD1 antibodies in monotherapy or combined with the CTLA-4 antibody ipilimumab or the LAG-3 antibody relatlimab have markedly improved survival outcomes [3,4,5,6]. This is achieved by blocking immune checkpoints that are expressed on T cells and downregulate immune activation [7]. By blocking these checkpoint receptors, activity against tumor cells is enhanced; however, this activation is not tumor-specific and can also trigger immune responses against healthy tissue, resulting in a broad spectrum of immune-related adverse events (irAEs) [8,9,10].

Gastrointestinal irAEs are among the most frequent toxicities associated with ipilimumab (42%) or the combination of ipilimumab plus nivolumab (46%) compared to nivolumab monotherapy (18%), with diarrhea and colitis being the most common manifestations [11]. High-grade diarrhea and colitis are major causes of immunotherapy discontinuation and can be life-threatening if not managed appropriately [12,13]. Most patients with severe diarrhea respond to oral or intravenous high-dose corticosteroids, but one-third to two-thirds of patients require additional systemic immunosuppression, most commonly with infliximab (5 mg/kg) [9,14].

Infliximab, a chimeric monoclonal antibody against tumor necrosis factor-α (TNF), is an established treatment of inflammatory bowel disease (IBD) [15]. Mycophenolate mofetil (MMF) is a relatively selective inhibitor of T- and B-lymphocyte proliferation [16]. Beyond its role in transplantation medicine, MMF has demonstrated beneficial effects in various autoimmune disorders and is already being used, in addition to corticosteroids, for severe ICI-induced pneumonitis or hepatitis [17]. In murine colitis models, MMF pretreatment reduced mortality and body weight loss and improved histopathological signs of inflammation, including decreased T-cell infiltration and reduced expression of interferon-γ, TNF-α, interleukin-4, and interleukin-10 [18]. A retrospective case series and review showed the impact of MMF in IBD patients resistant to azathioprine and infliximab by improving corticosteroid-free remission [19].

MMF has not yet been evaluated as a treatment alternative to infliximab in patients with ICI-induced severe ir diarrhea and ir colitis resistant to high-dose corticosteroids, although it could be a low-cost, well-tolerated oral drug alternative. In this study, we analyzed 52 melanoma patients with severe ipilimumab-induced colitis refractory to high-dose corticosteroids. Therapy escalation consisted of oral MMF in 31 patients and intravenous infliximab in 21 patients.

## 2. Materials and Methods

This is a multicenter, retrospective analysis comparing MMF and infliximab for the treatment of severe ir colitis in patients with advanced melanoma who had undergone immunotherapy with ipilimumab (10 mg/kg; 3 mg/kg), ipilimumab (3 mg/kg) plus nivolumab (1 mg/kg), or ipilimumab (3 mg/kg) plus vemurafenib (240 mg; 4-0-4/d) and who developed diarrhea as an irAE during treatment at the Skin Cancer Center in Heidelberg between May 2011 and May 2025, as well as at the Skin Cancer Centers in Hannover, Mainz, and Kiel. Patients who received high-dose systemic corticosteroids (≥1 mg/kg body weight prednisolone equivalent) for at least 24 h without any clinical improvement were classified as steroid-refractory. Steroid-refractory patients received, in addition to corticosteroids, either oral MMF (3 g per day) or intravenous infliximab (5 mg/kg body weight). In some patients, a second dose of infliximab was administered after one week. Patients were excluded if they had previously received immunotherapy with ipilimumab or ipilimumab plus nivolumab and had developed diarrhea as an irAE or if they had been treated with oral or intravenous high-dose corticosteroids for less than 24 h before escalation of immunosuppressive treatment with additional MMF or infliximab.

Medical records were reviewed to obtain patient data. Collected data included patient demographics, subsequent immunotherapies and irAEs, the start and end dates of ipilimumab treatment, the number of immunotherapy doses received, the date of tumor progression, and the reason for discontinuation of immunotherapy. Furthermore, we assessed the onset and resolution of diarrhea; the maximum stool frequency (grading according to CTCAE version 5.0); the treatment of diarrhea (including the start, dose and end of corticosteroids); the cumulative and peak steroid doses (all steroids were converted to prednisolone equivalents for comparability); the start, dose and end of additional immunosuppressive treatment; and if applicable the date of diarrhea recurrence. Additional data collected included cytomegalovirus (CMV) status after recurrence of diarrhea, bacterial stool cultures, the date and findings of the colonoscopy, calprotectin levels before and after diarrhea onset, LDH levels at the start of immunotherapy, and the date of death or last follow-up.

For calprotectin levels, we dichotomized them into <1800 µg/g and ≥1800 µg/g for statistical analyses since all patients showed elevated calprotectin levels after diarrhea onset and more than half of the cohort had levels above 1800 µg/g, which is the upper level given by the laboratory at Heidelberg University Hospital. LDH levels were standardized for statistical analyses such that values > 1 indicated elevated LDH, whereas values < 1 were regarded as normal.

Primary outcome measures were the response rate and the time to response during treatment with additional MMF or infliximab. Secondary outcome measures included the peak steroid dose, the duration of steroid intake, the cumulative steroid dose, the recurrence rate of diarrhea under both therapies, CMV positivity after recurrence of colitis, as well as PFS and OS measured from the initiation of corticosteroid treatment.

### Statistical Analysis

Patient characteristics were analyzed using descriptive statistics. The Kaplan–Meier method and the log-rank test were applied to estimate the time to response. Patients who did not experience stool normalization under their primary additional immunosuppression with MMF or infliximab were censored at the date of immunosuppressant change. The response rate, the recurrence rate, CMV positivity after recurrence of colitis, sex and the type of immunotherapy distribution were compared using Pearson’s chi-squared test. Differences in age between the two immunosuppressive groups were assessed using a *t*-test. The Mann- Whitney U test was used to compare baseline characteristics between the MMF and infliximab groups, including the type of immunotherapy, the number of immunotherapy cycles until the onset of diarrhea, the time from initiation of immunotherapy to diarrhea onset, the severity of diarrhea, the duration of corticosteroid treatment prior to initiation of MMF or infliximab, and the peak steroid dose.

The overall duration of corticosteroid intake was analyzed with the Kaplan–Meier method and the log-rank test. Patients who were still receiving corticosteroids at the end of the observation period or who died before corticosteroid tapering was completed were censored at the date of the last follow-up or death. PFS and OS in the MMF and infliximab groups, measured from the start of corticosteroid intake, were also estimated with the Kaplan–Meier method and log-rank tests. Patients who were alive at the last follow-up were censored at the date of the last follow-up for OS analysis, and patients without tumor progression after immunotherapy were censored at the date of the last follow-up for PFS analysis. Three patients in the MMF group who experienced disease progression before initiation of corticosteroid therapy under ICI treatment were excluded from the PFS analysis starting from corticosteroid initiation.

Pearson’s chi-squared tests were used to compare LDH levels at the start of immunotherapy and markedly elevated calprotectin levels after diarrhea onset between the MMF and infliximab groups. The Mann–Whitney U test was applied to compare the cumulative steroid dose distribution between the MMF and infliximab groups. The cumulative steroid dose distributions in the MMF and infliximab groups were illustrated using boxplots. In addition, the cumulative corticosteroid dose and the total duration of corticosteroid therapy were compared using the Mann–Whitney U test. These comparisons were performed within the infliximab group between the Heidelberg center and the other participating centers (Hannover, Mainz, and Kiel), as well as within the Heidelberg center between patients treated with MMF and those treated with infliximab. We applied Cox regression to assess the impact of cumulative steroid dose on PFS and OS. Furthermore, we evaluated whether cumulative steroid dose and the type of additional immunosuppressant (MMF or infliximab) influenced PFS or OS.

All statistical analyses were performed using SPSS Statistics version 27 (IBM Corp., Armonk, NY, USA). A two-sided significance level of 5% was applied.

## 3. Results

### 3.1. Patients

Between May 2011 and May 2025, 166 patients with melanoma developed ir diarrhea during ICI treatment with ipilimumab-containing regimens at the Skin Cancer Center in Heidelberg. A total of 17 patients (10%) had grade 1 diarrhea, while 61 patients had (37%) grade 2 diarrhea, and 88 patients (53%) had grade 3 diarrhea according to CTCAE v5.0. Forty patients (24%) had steroid-refractory diarrhea and required additional immunosuppression (nine treated with infliximab and 31 with MMF). To increase the number of infliximab-treated patients for comparison, we additionally included 12 infliximab-treated patients from the Skin Cancer Centers in Hannover, Mainz and Kiel.

A total of 46% of the included patients were male, and the median age at the start of immunotherapy was 61 years (range: 23–83 years). Patients received a median of two cycles of immunotherapy. Only five patients completed ICI treatment, while 47 discontinued treatment due to adverse events. Forty-four patients developed grade 3 diarrhea, and seven patients developed grade 2 diarrhea (CTCAE v5.0). Calprotectin levels were measured in 22 patients before the onset of diarrhea, with seven showing elevated levels. After the onset of diarrhea, calprotectin levels were measured in 39 patients, all of whom showed elevated values. Stool ruled out infectious causes at diarrhea onset. One patient tested positive for *Clostridium difficile* 55 days after onset. Nevertheless, she received infliximab ten days later, achieved stool normalization, and did not require specific treatment for *C. difficile* (Pat. 5, Table S2). Endoscopic evaluation (rectoscopy, sigmoidoscopy, or colonoscopy) was performed in 42 patients, of whom 41 showed findings consistent with ir colitis; one patient had inconclusive findings apart from erosive gastritis.

Twenty-two patients experienced additional irAEs, such as hypophysitis, pneumonitis, hepatitis, or pancreatitis (Tables S1 and S2). Eighteen patients underwent subsequent immunotherapy, five of whom developed recurrent diarrhea (Tables S1 and S2). LDH levels at the start of immunotherapy were measured in 50 patients, with 16 (32%) showing elevated levels.

No significant differences in baseline characteristics were observed between the MMF and infliximab groups (Table 1). However, there was a tendency toward a longer duration of corticosteroid treatment prior to infliximab compared with MMF (*p* = 0.091).

### 3.2. Ir Colitis Management

Twenty-four of 31 patients (77.4%) experienced normalization of stool frequency under additional MMF after a median of seven days (range: 2–56 days). The median duration of MMF intake was 73 days. Seven patients required infliximab after failure of MMF after a median of 9 days (range: 3–13 days). One patient initially received MMF; after recurrence of colitis, a single dose of infliximab was administered without achieving bowel habit normalization within 5 days. Re-administration of MMF then resulted in normalization after another 16 days.

Twenty of 21 patients (95.2%) achieved normalization of stool frequency with infliximab after a median of eleven days (range: 1–60 days). To achieve a resolution of symptoms, up to three infliximab infusions were given; however, the median number of infusions needed was one. One patient was successfully treated with MMF 24 days after failure of two infliximab infusions. After initiation of MMF, bowel habit normalization was achieved after six days (Pat. 2, Table S2).

**Figure 1:**
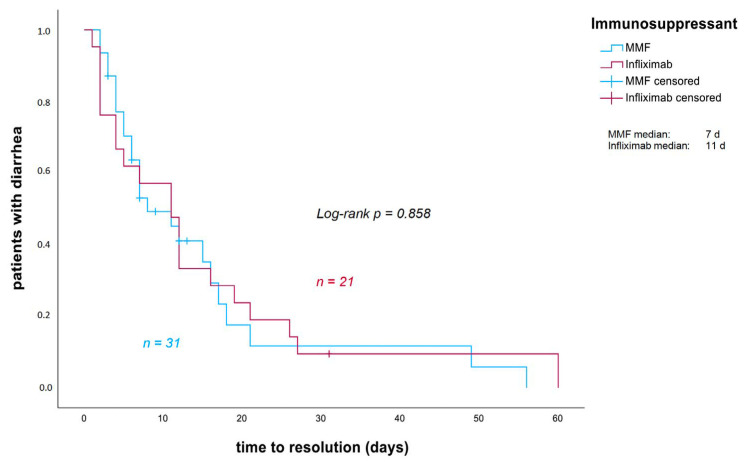
Time to resolution of diarrhea after the start of MMF/Infliximab in days. Patients who did not experience stool normalization while undergoing their primary additional immunosuppression with MMF or infliximab were censored at the date of immunosuppressant change. MMF: mycophenolate mofetil.

Response rates to MMF or infliximab (*p* = 0.081) and the time to response (*p* = 0.858) did not differ significantly between the two groups (Figure 1).

**Figure 2:**
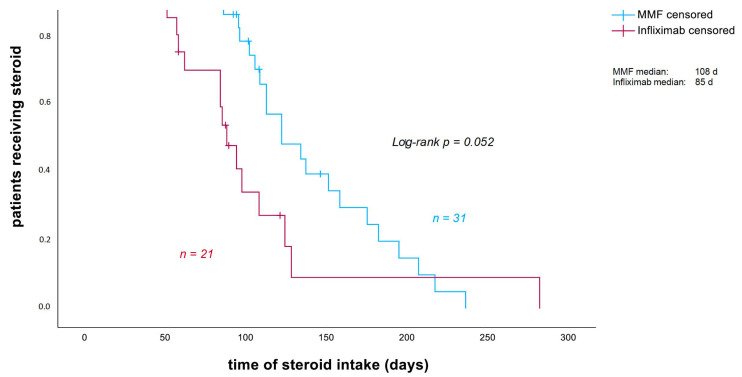
Duration of corticosteroid intake (in days). Patients who still received steroids at the end of the observation period or died before weaning were censored at the date of the last follow-up or the date of death. MMF: mycophenolate mofetil.

**Figure 3:**
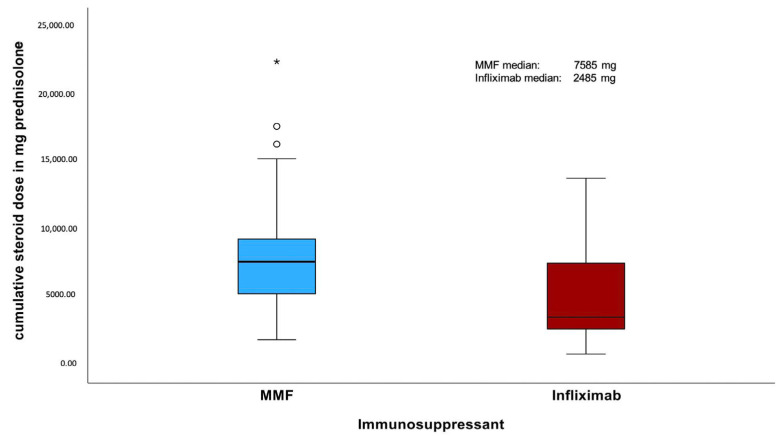
Boxplot of cumulative steroid dose (mg prednisolone equivalent) by type of additional immunosuppressive therapy (MMF vs. Infliximab). Small hollow circles (○) indicate outliers; the asterisk (*) represents an extreme outlier. MMF: mycophenolate mofetil.

The median duration of corticosteroid intake in patients with severe ir colitis was 108 days in the MMF group, compared to 85 days in the infliximab group. This shows a tendency towards a shorter corticosteroid treatment duration in patients treated with infliximab (*p* = 0.052; Figure 2). The cumulative steroid dose was retrospectively assessed in 51 of 52 patients. The dose could not be determined in one patient, as steroid tapering was carried out externally without sufficient documentation. The median cumulative steroid dose was significantly higher in the MMF group (7585 mg) compared to the infliximab group (3485 mg) (*p* = 0.002; Figure 3).

Within the infliximab group, patients treated at the Heidelberg center (*n* = 9) had a significantly longer total duration of corticosteroid therapy than patients treated at the other participating centers (*n* = 12; median 89 vs. 58 days; *p* = 0.039). Likewise, the cumulative corticosteroid dose was significantly higher in infliximab-treated patients from Heidelberg (*n* = 8) compared with those treated at the centers in Hannover, Mainz, and Kiel (*n* = 12; median 7486.25 vs. 2711 mg; *p* = 0.002).

Within the Heidelberg center, no significant differences were observed between patients treated with MMF and those treated with infliximab regarding the total duration of corticosteroid therapy (median 108 vs. 89 days; *p* = 0.206) or the cumulative corticosteroid dose (median 7585 vs. 7486.25 mg; *p* = 0.835).

Peak steroid dose was assessed in all patients and was comparable between both groups, with a median of 2.5 mg/kg BW (range 1–5) in the MMF group and a median of 2.5 mg/kg BW (range 0.81–4.29) in the infliximab group, showing no significant difference (*p* = 0.345; Table 1).

### 3.3. Recurrences of Colitis

Seven of 31 patients (22.6%) experienced recurrence of colitis under MMF treatment after a median of 38 (range 17–59) days since MMF treatment initiation. Two of these patients (28.6%) undergoing treatment with MMF tested positive for CMV based on rectal biopsy. One of these patients (pat. No. 5, Table S1) received additional infliximab 26 days prior to the positive CMV polymerase chain reaction (PCR). In the infliximab group, four of 21 patients (19%) had recurrence of colitis after a median of 32.5 (range 30–56) days, with one patient (25%) testing positive for CMV 39 days after infliximab infusion. No further infections were reported in patients receiving multiple lines of immunosuppression.

Neither recurrence rates after additional immunosuppression (*p* = 0.760) nor CMV positivity at recurrence of colitis (*p* = 0.898) differed between the groups.

### 3.4. Oncological Outcome

**Figure 4:**
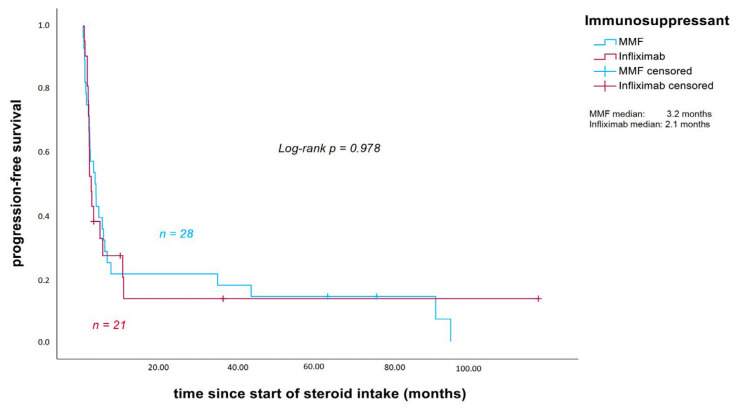
Progression-free survival measured from the start of corticosteroid intake (in months). Patients without disease progression were censored at the date of the last follow-up. Three patients were excluded because they experienced progression of disease before corticosteroid intake. MMF: mycophenolate mofetil.

**Figure 5:**
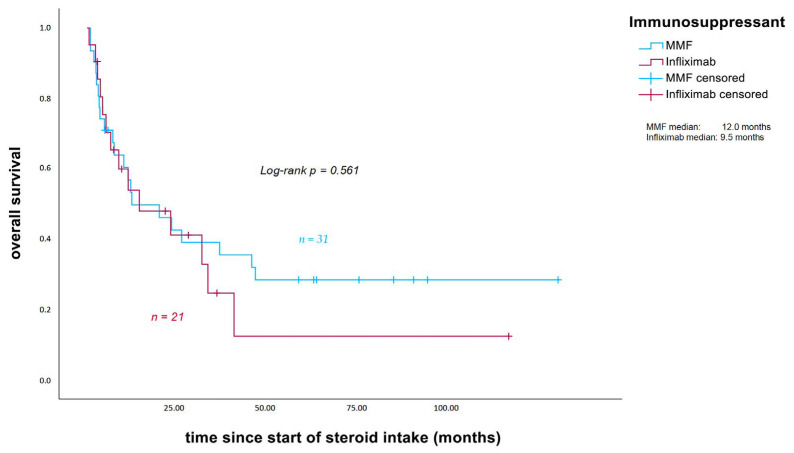
Overall survival measured from the start of corticosteroid intake (in months). Patients who survived were censored at the date of the last follow-up. MMF: mycophenolate mofetil.

Median PFS, measured from the start of corticosteroid intake, was 3.2 months in patients receiving additional MMF (calculated for 28 patients) and 2.1 months in those treated with infliximab (*p* = 0.978; Figure 4). Median OS was 12 months in the MMF group and 9.5 months in the infliximab group (*p* = 0.561; Figure 5).

In addition, the cumulative steroid dose did not have a statistically significant impact on PFS (*p* = 0.447) or OS (*p* = 0.270), measured from the start of corticosteroid intake. Even when analyzed as a covariate with additional immunosuppression (MMF or infliximab), cumulative steroid dose showed no significant influence on PFS (*p* = 0.731) or OS (*p* = 0.541).

## 4. Discussion

To date, only few studies have investigated alternative treatment options apart from standard recommendations for patients with irAEs. While MMF is considered the drug of choice in ICI-induced ir hepatitis, its use in ICI-induced ir colitis remains uncommon [17]. In this retrospective analysis, we demonstrate that patients with advanced melanoma and ICI-induced ir colitis refractory to high-dose corticosteroids can be successfully managed not only with infliximab but also with MMF. No statistically significant differences were observed in the response rate of diarrhea or the time to response, although infliximab showed a tendency towards higher efficacy (95.2% vs. 77.4%, *p* = 0.081). Seven patients who initially received MMF for steroid-refractory colitis required additional treatment with infliximab. All of them achieved stool normalization after a single infliximab infusion, without recurrence of colitis, and tolerated multiple lines of immunosuppression without significant adverse effects. In a prospective study in patients with treatment-naive metastatic melanoma and ICI-induced grade 3 ir colitis, the addition of 1 g MMF twice daily to 2 mg/kg intravenous methylprednisolone upfront was evaluated. Eleven patients were treated with this regimen, achieving a median time to patient-reported normalization of bowel habit of 10 days. Four patients (36%) developed a colitis flare, which was successfully managed with infliximab (5 mg/kg; one infusion in three patients and two infusions in one patient). All patients were successfully weaned from corticosteroids, and none experienced recurrence within 8 weeks after discontinuation of MMF. The authors concluded that upfront MMF in combination with high-dose steroids may reduce the need for corticosteroid escalation or additional infliximab, decrease the risk of recurrence during tapering, and potentially accelerate symptom resolution [20]. Comparison with our results is limited, as our cohort consisted of patients with steroid-refractory colitis, likely reflecting more severe diseases. Nevertheless, the median time to bowel normalization and the response rates in our analysis were broadly comparable, though slightly more favorable for MMF [20].

No statistically significant differences in oncological outcomes (PFS and OS, measured from the start of corticosteroid intake) were observed between patients treated with MMF and those treated with infliximab, although outcomes seemed slightly more favorable in the MMF group (median PFS 3.2 vs. 2.1 months, *p* = 0.978; median OS 12 vs. 9.5 months; *p* = 0.561), despite a tendency towards a longer treatment duration and a significantly higher median cumulative steroid dose in patients who initially received MMF.

Survival outcomes following anti-TNF therapy have been reported inconsistently. In a large cohort of 1250 patients with advanced melanoma treated with first-line ipilimumab, pembrolizumab, or ipilimumab plus nivolumab, anti-TNF therapy given for severe toxicities, most commonly colitis, was associated with significantly shorter survival compared with using steroids alone [21]. Consistent with our observations, data from the Side Effect Registry Immuno-Oncology (SERIO) indicated slightly better PFS in patients receiving additional MMF compared with infliximab, although infliximab was mainly administered as second-line therapy in ir colitis, whereas MMF was primarily used in ir hepatitis [22]. In contrast, preclinical studies suggest potential benefits of anti-TNF. In murine models of ipilimumab plus nivolumab, the addition of anti-TNF improved tumor control and survival compared with checkpoint blockade alone [23]. Similar results were reported when PD-1 blockade was combined with anti-TNF, showing superior tumor regression and survival compared with PD-1 inhibition alone [24]. A retrospective analysis further suggested improved OS in patients with ICI-induced ir colitis who received early infliximab and at least two infusions in addition to steroids [25].

Patients treated with infliximab as additional immunosuppressive therapy showed a tendency towards a shorter duration and a significant lower cumulative intake of corticosteroids compared with those receiving MMF (85 vs. 108 days, Figure 2; 3485 vs. 7585 mg prednisolone, Figure 3). Within the infliximab cohort, patients treated at the Heidelberg center had both a significantly longer duration of corticosteroid therapy and a higher cumulative dose than patients treated at the other participating centers. In contrast, no significant differences in corticosteroid duration or cumulative dose were observed in the center-internal comparison between MMF- and infliximab-treated patients at Heidelberg.

These subgroup analyses may indicate center-dependent differences in the initiation and tapering of corticosteroid therapy, as all MMF-treated patients were managed in Heidelberg, whereas infliximab-treated patients were predominantly treated at other institutions. Accordingly, the observed differences in corticosteroid exposure are more likely related to center-specific management strategies than to substance-specific effects. Given the retrospective design and the absence of a standardized corticosteroid tapering protocol, it cannot be determined whether MMF inherently requires longer or higher corticosteroid exposure compared with infliximab to achieve comparable clinical effectiveness. However, the prolonged corticosteroid intake and higher cumulative steroid dose in the MMF group did not impact the clinical outcome of melanoma patients. This is in line with a recent pooled analysis of six studies including almost 2000 patients receiving combination treatment of ipilimumab plus nivolumab across tumor types [26]. However, an earlier retrospective study reported a negative impact of cumulative steroid doses (>4000 mg) on ICI efficacy in patients with ir hepatitis [27]. In contrast, a review of 27 studies found no evidence that corticosteroids given for the management of irAEs reduce the efficacy of immunotherapy and was unable to define a dose or exposure threshold above which efficacy would be compromised [28]. Taken together, current evidence suggests that the peak dose of corticosteroids during ICI therapy—rather than the cumulative dose—may influence antitumor efficacy; in our study, peak steroid doses were similar between both groups [26].

Baseline characteristics showed a tendency towards a longer duration of corticosteroid intake prior to the initiation of infliximab compared with MMF (median 20 vs. 15 days, *p* = 0.091), supporting the notion that infliximab is used more cautiously in routine clinical practice.

### Limitations

This study represents a retrospective analysis of data collected in routine clinical practice. Data quality therefore depended on clinical documentation, as no standardized study protocols were available. Likewise, treatment regimens were not uniform, and the decision to initiate corticosteroids or additional immunosuppression, as well as the dosage used, was based on the clinical assessment of the treating physicians. Given the multicenter design, variations in the management of adverse events between institutions are likely, which may have impacted the timing of initiation of second-line immunosuppression, the duration of steroid treatment, and the cumulative steroid dose. Interpretation of clinical data may also have differed across centers, as documentation was not reviewed by a single assessor. Finally, the relatively small patient cohort limits the statistical power of the analyses.

## 5. Conclusions

Our study demonstrates that patients with steroid-refractory, ICI-induced ir colitis can be effectively managed not only with infliximab but also with MMF, without prolongation of time to resolution or impairment of oncological outcomes. The response rate seems slightly better when treated with infliximab, whereas the time to resolution seems slightly more favorable when treated with MMF. While MMF was associated with a tendency towards longer corticosteroid intake, the significantly higher cumulative steroid dose in those undergoing MMF treatment had no significant impact on progression-free or overall survival. Also, the observed differences in corticosteroid exposure are more likely related to center-specific management strategies than to substance-specific effects. Given its oral administration, good tolerability, and lower cost, MMF may represent a viable alternative for patients in whom infliximab is contraindicated or less feasible. Prospective studies are warranted to further define the role of MMF in the management of ICI-induced ir colitis.

## Figures and Tables

**Table 1:** Baseline characteristics of patients treated with mycophenolate mofetil (MMF) or infliximab.

	MMF (*n* = 31)	Infliximab (*n* = 21)	*p*-Value
**Sex**			0.862
male	14 (45.2%)	10 (47.6%)	
female	17 (54.8%)	11 (52.4%)	
**Age** (years)	62 (33–83)	60 (23–80)	0.793
**Immunotherapy**			0.597
Ipilimumab monotherapy	12 (38.7%)	10 (47.6%)	
Ipilimumab + nivolumab	18 (58.1%)	11 (52.4%)	
Ipilimumab + vemurafenib	1 (3.2%)		
**LDH Level at the start of immunotherapy**			
Elevated levels	8/30 (26.7%)	8/20 (40%)	0.322
**Immunotherapy cycles until diarrhea onset**	2 (1–4)	2 (1–5)	0.547
**Time to the onset of diarrhea** (days)	34 (3–90)	29 (2–73)	0.121
**Severity of diarrhea** (CTCAE v5.0)	3 (2–3)	3 (2–3)	0.539
**Calprotectin at the start of diarrhea**			
Elevated level	24/24 (100%)	15/15 (100%)	
≥1800 µg/g	15/24 (62.5%)	10/15 (66.7%)	0.792
**Time of corticosteroid treatment prior to MMF/infliximab** (days)	15 (1–80)	20 (4–172)	0.091
**Peak steroid dose**			
(in mg/kg BW prednisone-equivalent)	2.5 (1–5)	2.5 (0.81–4.29)	0.345

Data are presented as absolute numbers and percentages. Bold text indicates overarching category headings. Age, immunotherapy cycles until diarrhea onset, time to the onset of diarrhea, severity of diarrhea, time to corticosteroid treatment prior to MMF/infliximab and peak steroid dose are given as medians and ranges. *p*-values refer to comparisons between treatment groups. BW: body weight, CTCAE v5.0: Common Terminology Criteria for Adverse Events Version 5.0, LDH: lactate dehydrogenase, MMF: mycophenolate mofetil.

## Data Availability

The data underlying this study were extracted from internal clinical information systems and compiled into an anonymized Excel database for analysis. Due to institutional and patient privacy regulations, the dataset cannot be made publicly available. Data may be provided by the corresponding author upon reasonable request and with permission from the responsible institution.

## Supplementary Materials

The following supporting information can be downloaded at https://www.mdpi.com/article/10.3390/cancers18050734/s1, Table S1: Individual patient characteristics and clinical course of patients treated with mycophenolate mofetil (MMF) for steroid-refractory immune-related colitis.; Table S2: Individual patient characteristics and clinical course of patients treated with infliximab for steroid-refractory immune-related colitis.

## Abbreviations

The following abbreviations are used in this manuscript:

Anti-PD-1	Anti-Programmed Death 1
BW	Body Weight
CMV	Cytomegalovirus
CTCAE	Common Terminology Criteria for Adverse Events
CTLA-4	Cytotoxic T-Lymphocyte Antigen 4
DKFZ	Deutsches Krebsforschungszentrum
IBD	Inflammatory Bowel Disease
ICI	Immune Checkpoint Inhibitor
irAE	Immune-related adverse event
ir-colitis	Immune-related colitis
LDH	Lactate Dehydrogenase
LAG-3	Lymphocyte Activation Gene 3
MMF	Mycophenolate mofetil
NCT	National Center for Tumor Disease
OS	Overall Survival
PCR	Polymerase Chain Reaction
PFS	Progression-Free Survival
SPSS	Statistical Package for the Social Sciences
TNF	Tumor Necrosis Factor
TNF-a	Tumor Necrosis Factor alpha
