# Supplementary material for: Mycophenolate Mofetil for Treatment of Ipilimumab-Induced Colitis in Patients with Metastatic Melanoma
Source: Cancers (Basel). 2026 Feb 25;18(5):734. doi: 10.3390/cancers18050734 (PMC12985017; doi:10.3390/cancers18050734)
Supplement: Supplementary file 1 [file cancers-18-00734-s001.zip › cancers-4135409-supplementary.pdf]

Pat	Age	Sex	Center	Treatment intention	Treatment line	ICI	ICI-cycles	Best response	Grade of diarrhea	Colonoscopy	Time of steroid intake prior to MMF (days)	Time of steroid intake (days)	Cumulative steroid dose	Peak steroid dose	MMF-intake (days)	+ Infliximab	Failure of MMF	Time to resolution (days)[1]	Recurrence of colitis after MMF (in days)	CMV-+ at recurrence	Other irAE	FU-ICI	Diarrhea after FU-ICI
**1**	63	F	HD	p	1	I3	3	PD	3	+	16	158	8480	2.5	70	-	-	49	-		-	P	-
**2**	68	M	HD	p	1	IN	2	PR	3		3	95	6605	2.5	15	-	-	17	-		-	-	
**3**	53	M	HD	p	1	I3	2	PR	3	+	11	108	10552.5	2.5	10	+	+	24	-		-	P	-
**4**	65	M	HD	p	1	I3	4	PD	3	+	18	182	17603.8	2.5	56	+[2]	-	16	17	-	E;Hy	-	
**5**	70	M	HD	p	3	I10	2	PD	3		15	146	7240	1.11	130	-	-	5	43	-	-	-	
**6**	77	F	HD	p	1	IN	4	PD	3		43	102	5255	1	73	-	-	7	-		-	N	-
**7**	53	F	HD	p	1	IN	2	SD	3		19	236	10170	2.5	12	+	+	90	-		Pn,He	P	+
**8**	68	M	HD	p	1	I3	2	SD	3	+	3	207	15205	2.5	213	-	-	7	21	-	Hy	P	-
**9**	79	F	HD	p	2	I3	2	PR	2	+	15	54	1820	1	42	-	-	3	-		-	-	
**10**	55	M	HD	p	2	I3	3	PD	2	+	35	217	16285	2.8	66	-	-	18	-		-	P	+
**11**	33	F	HD	p	3	I(N)[3]	3	PR	3	+	80	122	9330	3.3	12	+	+	17	-		E	-	
**12**	78	F	HD	p	2	I3	3	PD	3	+	14	94	7060	2.5	79	-	-	3	38	+	E,Pr	-	
**13**	56	F	HD	p	2	IN	4	PD	3	+	15	28	3375	2.4	11	-	-	11	-		Pa,He	-	
**14**	66	M	HD	p	1	I(N)[4]	3	PD	3		10	28	4510	2.5	17	-	-	2	-		E	-	
**15**	67	F	HD	p	2	IN	2	PD	3	+	4	101	9211.3	2.5	97	+	+	11	26	+	-	-	
**16**	56	F	HD	p	3	IN	3	SD	2		47	122	5035	1	100	-	-	6	-		He,Th, Ne	-	
**17**	59	F	HD	p	1	IN	4	PR	3	-	5	175	5850	1.25	198	-	-	4	-		Hy,Pa,Ga	-	
**18**	52	M	HD	a	1	I3	2	PR	3	+	53	195	22389.5	5	6	+	+	14	-		He	P	-
**19**	48	F	HD	p	3	IV	3	PD	3	+	1	113	8977.5	2.5	89	-	-	21	-		-	-	
**20**	76	M	HD	p	1	IN	1	PD	3	+	7	108	6560	2.5	101	-	-	7	59	-	-	-	
**21**	51	F	HD	p	1	I3	4	PD	3	+	13	96	8745	2.5	93	-	-	6	-		Oph	P	-
**22**	62	F	HD	p	2	IN	4	PD	3	+	19	92	6249	2.5	94	-	-	8	-		-	-	
**23**	64	F	HD	p	2	IN	2	PR	3	+	55	151	4615	2.5	89	-	-	15	-		-	N	+
**24**	83	M	HD	p	1	IN	2	PD	3		4	57	5190	2.5	53	-	-	5	-		-	-	
**25**	41	M	HD	p	1	IN	1	SD	3	+	6	137	9495	2.5	143	-	-	56	-		Neu	-	
**26**	73	M	HD	p	1	IN	2	CR	3	+	21	86	7595	2.5	71	-	-	4	-		Th,Pa	-	
**27**	42	F	HD	p	3	IN	2	PD	2	+	21	44	4320	1.25	53	-	-	12	-		Pa	-	
**28**	72	F	HD	a	3	I3	1	SD	3	+	6	128	8755	2.5	114	+	+	11	58	-	E	-	
**29**	57	F	HD	p	2	IN	2	PD	2	+	15	47	4400	2.5	31	-	-	2	-		-	-	
**30**	58	M	HD	p	2	IN	1	PD	3		33	105	8597.5	2.5	77	-	-	4	-		-	-	
**31**	59	M	HD	a	2	I3	2	PD	3	+	20	113	7585	2.5	117	+	+	12	-		E, Pa	IN	+

Table S1 Pat: patient; F: female; M: male; HD: Heidelberg; p: palliative; a: adjuvant; ICI: immune checkpoint inhibitor; I3: ipilimumab 3mg/kg body weight; I10: ipilimumab 10mg/kg body weight; IN: ipilimumab 3mg/kg plus nivolumab 1mg/kg body weight, IV: ipilimumab 3mg/kg body weight plus vemurafenib 240mg 4-0-4; CR: complete remission; PR: partial remission; SD: stable disease; PD: progressive disease; colonoscopy = blank cells indicate colonoscopy not performed, +: colonoscopy performed with ir-colitis finding, -: colonoscopy performed without ir-colitis finding; cumulative steroid dose in mg prednisolon equivalent; peak steroid dose in mg/kg body weight prednisolon equivalent; additional infliximab = +: infliximab was administered, -: infliximab was not administered; recurrence of colitis = +: patient experienced recurrence, -: patient did not experience recurrence; cmv pos. = blank cells indicate patients weren’t tested, +: patients were tested cmv positive, -: patients were tested cmv negative; other immune related adverse event (irAE)= E: eczema, Hy: hypophysitis, He: hepatitis, Pn: pneumonitis, Pr: pruritus, Pa: pancreatitis, Th: thyroiditis, Ne: nephritis, Ga: gastritis, Oph: ophtalmomyositis, Neu: neuropathy; follow-up immune checkpoint inhibitor (FU-ICI) = P: pembrolizumab, N: nivolumab, IN: ipilimumab plus nivolumab; diarrhea after FU-ICI = +: yes, -: no

[1] measured from date of first intake of MMF to date of stool frequency normalization.

[2] at recurrence a single dose of infliximab was administered without achieving bowel habit normalization within 5 days. Re-administration of MMF resulted in resolution of diarrhea after another 16 days.

[3] patient 11 received two doses of ipilimumab plus nivolumab and then one dose of ipilimumab 3mg/kg

[4] patient 14 received one dose of ipilimumab plus nivolumab and then two doses of ipilimumab 3mg/kg

Pat	Age	Sex	Center	Treatment intention	Treatment line	ICI	ICI-cycles	Best response	Grade of diarrhea	Colonoscopy	Time of steroid intake prior to Infliximab	Time of steroid intake (days)	Cumulative Steroid Dose	Peak steroid dose	Infliximab doses	+ MMF	Failure of infliximab	Time to resolution (days)[1]	Recurrence of colitis after infliximab (in days)	CMV-+ at recurrence	Other irAE	FU-ICI	Diarrhea after FU-ICI
**1**	62	F	HD	p	1	I3	2	SD	3	+	26	88	-	1	1	-	-	5	-		-	P	-
**2**	80	M	HD	p	2	I3	2	PD	2	+	39	87	9180	2.5	2	+	+	37	-		-	-	
**3**	59	M	HN	p	1	I3	3	PD	3		64	128	1450	1.09	1	-	-	60	-		-	P	-
**4**	51	F	HN	p	2	I3	2	PD	3	+	48	84	3150	0.81	1	-	-	19	-		-	-	
**5**	44	F	HN	p	1	I3	2	PD	3	+	25	57	3100	1.61	1	-	-	12	-		-	P	-
**6**	55	F	HN	p	1	I3	4	SD	3	+	37	108	4425	1.53	1	-	-	27	-		-	N	-
**7**	23	M	HN	a	1	I3	5	CR	3	+	17	124	2600	1.07	1	-	-	2	-		Pa	-	
**8**	57	F	HN	p	2	(I)N[2]	2	SD	3	+	16	32	1840	1.05	1	-	-	16	30	-	Th, Pa	-	
**9**	64	F	HN	p	2	IN	2	SD	3	+	19	58	2360	2.86	1	-	-	4	-		Th	-	
**10**	58	M	M	p	2	I3	1	PD	3	+	12	47	3500	2.5	2	-	-	2	-		-	-	
**11**	74	M	M	p	1	I3	3	PD	/[3]	+	4	51	2800	2.5	2	-	-	2	-		-	-	
**12**	72	M	M	p	2	IN	3	PD	3	+	8	16	760	2	1	-	-	2	-		Th	-	
**13**	60	F	M	p	2	IN	2	PR	2	+	11	58	2622.5	2	3	-	-	4	34	-	-	N	-
**14**	66	F	K	a	2	I3	3	CR	3	+	8	84	8798.4	4.29	1	-	-	12	31	+	-	-	
**15**	73	M	HD	p	1	IN	3	PD	3	+	51	121	13765	2.5	2	-	-	12	-		He, Hy	-	
**16**	54	F	HD	p	1	IN	1	PD	3	+	14	94	6045	2.5	1	-	-	11	-		-	-	
**17**	60	M	HD	p	1	IN	2	CR	3	+	17	89	5865	2.5	2	-	-	26	56	-	-	-	
**18**	71	F	HD	p	1	IN	3	PD	3	+	20	62	3470	2.5	1	-	-	21	-		-	-	
**19**	63	F	HD	a	2	IN	1	SD	3	+	172	282	7397.5	2.5	1	-	-	11	-		-	N	+
**20**	55	M	HD	p	1	IN	2	PD	3		25	85	7735	2.5	1	-	-	7	-		E	P	-
**21**	70	M	HD	p	1	IN	1	PD	3	+	22	97	7575	3.13	1	-	-	1	-		-	N	-

Table S2 Pat: patient; F: female; M: male; HD: Heidelberg, HN: Hannover, M: Mainz, K: Kiel; ICI: immune checkpoint inhibitor; I3: ipilimumab 3mg/kg body weight; I10: ipilimumab 10mg/kg body weight; IN: ipilimumab 3mg/kg body weight plus nivolumab 1mg/kg body weight; CR: complete remission; PR: partial remission; SD: stable disease; PD: progressive disease; colonoscopy = blank cells indicate colonoscopy not performed, +: colonoscopy performed with ir-colitis finding, -: colonoscopy performed without ir-colitis finding; cumulative steroid dose in mg prednisolon equivalent; peak steroid dose in mg/kg body weight prednisolon equivalent; additional MMF = +: MMF was administered, -: MMF was not administered; recurrence of colitis = +: patient experienced recurrence, -: patient did not experience recurrence; cmv pos. = blanke cells indicate patients weren't tested, +: patients were tested cmv positive, -: patients were tested cmv negative; other immune related adverse event (irAE)= E: eczema, Hy: hypophysitis, He: hepatitis, Pa: pancreatitis, Th: thyroiditis; follow-up immune checkpoint inhibitor (FU-ICI)= P: pembrolizumab, N: nivolumab, IN: ipilimumab plus nivolumab; diarrhea after FU-ICI= +: yes, -: no

[1] measured from date of first dose of infliximab to date of stool frequency normalization.

[2] patient 8 received two doses of ipilimumab plus nivolumab and then two doses of nivolumab 1mg/kg

[3] unknown frequency because patient has an anus praeter
